# Drs. Elliotson, Dickson, and Martinet on Quinine and Sulphate of Quinine

**Published:** 1824-09-01

**Authors:** 


1. *Illustrations of the Medical Properties of Quinina.* By JOHN ELLIOTSON, M.D. Fellow of the Royal College of Physicians, and Physician to St. Thomas's Hospital.
[Med. Chir. Trans. Vol. XII.]

2. *On the Febrifuge Power of the Sulphate of Quinine.* By D. J. H. DICKSON, M. D. Physician to the Royal Naval Hospital, Plymouth.
[Ed. Med. Journal, Oct. 1823.]

3. *Sur l'Emploi du Sulfate de Quinine à Haute Dose dans des Cas de Fièvres Intermittentes, observées in Italie.* Par L. MARTINET, M.D.

[Revue Medicale.]

Of all the energetic substances which chemical ingenuity has lately extracted from different articles of the materia medica, those from cinchona appear to us most likely to be permanently and highly useful. We have already seen such powerful and salutary effects from the substance at the head of this paper, that we cannot help anticipating from it the most beneficial results to therapeutical medicine.

Dr. Elliotson, a most able and zealous cultivator of his profession, was among the first in this country to draw the attention of the faculty to the remedy under review, in a paper read at the Medico-Chirurgical Society, on the 8th July 1823, and since published in the last volume of their Transactions. Since that period, several papers have appeared in periodical journals, containing detached cases of the exhibition of the medicine of which we occasionally took notice in our Periscope.

M. Pelletier, about four years ago, furnished M. Magendie with specimens of the cinchonine alcalies, and this ingenious experimentalist soon proved them to be innocent, and applicable to remedial purposes. M. Chomel, at La Charité, was among the first to exhibit the sulphate of quinine in cases of intermittent fever, and with success. This practice has been now extensively followed on the Continent, and although some injurious effects have followed large doses of the medicine, its good character daily and hourly improves. But it is not to agues alone that its administration may be confined. In all the periodical complaints, for which bark and arsenic have been formerly prescribed, the sulphate of quinine will prove a valuable substitute in as small a form as can well be wished. In various anomalous and cachectic diseases, we have reason to hope much from this powerful tonic.

Among the first cases in which Dr. Elliotson prescribed this remedy, was one of typhus fever, brought to St. Thomas's Hospital. The patient was a poor half-starved Irish woman. She was supported by beef-tea and milk—the epigastrium, forehead, and occiput, were blistered, and hydrargyrus cum creta was administered in doses of one to two scruples every six hours, till the mouth became sore. The delirium and stupor were entirely subdued—the tongue became clean and moist—but debility increased hourly. The face became ghastly, and the body sunk lower in the bed. Dr. E. ordered three, and soon, five grains of the sulphate of quinina every six hours, the diet to continue the same. "A striking amendment was observed the next day, and she speedily recovered." When about to be discharged, she suddenly relapsed into great prostration of strength, but the quinina again restored her to strength. Dr. Elliotson observes that, although in doses of five grains every six hours, he never observed any bad effects from the sulphate, yet a dose of ten grains occasioned vomiting in the only three instances where he ventured on such a quantity. Dr. E. next relates three cases of intermittent fever, treated at St. Thomas's Hospital with the sulphate of quinina. The first was a quartan, of long standing. He was ordered five grains of the sulphate every six hours. The first paroxysm was mitigated, and no more returned. The second case was a tertian, of short duration. Only one paroxysm occurred after the commencement of the sulphate. The third case proved more refractory; but the disease felt the influence of the medicine almost immediately, and was removed in little more than a fortnight.

Conceiving that the virtue of the medicine must necessarily reside in the quinina itself, which, though impure, might be procured at a much less expence than the sulphate; Dr. Elliotson had some prepared in the following manner.

"The article was prepared for my use, by digesting cinchona in a very dilute solution of sulphuric acid (℥ij to four gallons of water) straining, and then adding magnesia to saturation, by which means the quinina was precipitated from the acid, mixed with tannin and extractive matter, and sulphate of magnesia remained in solution. The precipitate was again dissolved in sulphuric acid, again precipitated, and finally washed and dried. A pound of cinchona cordifolia furnishes about an ounce of this impure quinina, or about two drams of pure sulphate of quinina by another process, in which the quinina is obtained pure, by means of alcohol previously to its formation into a sulphate, whence the greater real expence of this article."—*Med. Chir. Trans.* Vol. xii, Part ii. p. 554.

Although some of the cases treated with the quinina alone are less decisive than others, yet, as far as they go, they all shew the power of the medicine, without exception. That failures will occur with any remedy, is but too certain, for, as Dr. E. justly observes, "no remedy is so specific but that the previous removal or diminution of some morbid, or at least, unfavorable condition, may be requisite to its success." Eleven cases of intermittent fever treated with the quinina are related by Dr. Elliotson, and all of them were cured in a moderate, some in a very short time. The dose was generally, we might say always, five grains every six hours. "My general experience," says Dr. E. "of simple quinina, as a tonic, is the same as of the sulphate; but I have never observed derangement of the stomach induced by doses of the impure preparation, I employed, so large as ten grains, every six hours." He begs, however, not to be understood as recommending simple quinina, in preference to the sulphate. His object is merely to illustrate the virtues of the substance, whether simple or combined. Several of Dr. E's friends have employed the sulphate with success. It succeeded in cases where the bark had previously failed. We shall adduce the following case, furnished by Dr. Roots.

"M. Sullivan was admitted into St. Pancras Infirmary, on May 7th, and had been suffering under tertian for nearly a month. She took the liquor arsenicalis from the 8th of May to the 23d, and every two or three days, rhubarb and calomel, without any advantage. From the 23d of May to June 6th, she took cinchona in drachm doses every six hours, with the decoction and tincture, continuing at times the rhubarb and calomel. As the paroxysms still returned at the regular period, the cinchona was discontinued, and the liquor arsenicalis resumed in doses of ♏ ix, every six hours, which she took from the 6th of June till the 20th. On the 20th, finding the paroxysms still return at the usual period, I ordered her five grains of the sulphate of quinina, in pill, to be given every six hours.

"'She took twelve doses, never having any return of the paroxysm after the first dose, and was discharged on the 2nd of July.

"'It is right to mention, that the day prior to the sulphate of quinina being ordered, she was allowed a pint of porter daily.'

"Dr. Roots, I may add, has employed the medicine but once besides, and says, in his letter to me, 'In another case of quartan, the sulphate was given in doses of two grains every six hours, and was equally successful.'" 560.

Dr. Elliotson has never observed any bad effects from the sulphate or simple quinina—except what has been stated, viz. sickness of stomach, when the dose was augmented to ten grains. He properly observes that—" quantities that can disagree are not required." Five grains of the sulphate every six hours, he thinks, is the largest dose that can be necessary. We have not found it necessary to give more than twelve or fourteen grains in the twenty-four hours to adults. The medicine, in all cases, should be continued one or two weeks after the symptoms have ceased.

It is true, as Dr. Elliotson remarks, that these are not *new* remedies, since we have been prescribing them ever since bark came from South America. But they are new forms—they are, in short, *the remedy* freed from a huge envelop of inert and disgusting matter.* Every body knows how we were obliged to "*throw in*" the bark in intermittent and remittent fevers—not seldom with the effect of overloading the stomach, and causing it all to be *thrown out* again. The astonishing power of a few grains of these active principles over the orgasms of fever, may be compared (as Dr. E. has aptly done) to the effect of throwing a little dust upon bees engaged in battle, as described in the Georgics of Virgil.

————————Hæc certamina tanta,
*Pulveris exigui* jactu, compresse quiescunt.

Dr. Dickson (then of Clifton) has given his testimony in favour of the febrifuge qualities of the sulphate of quinine. He first tried it in the case of one of his own children, five years of age, who had been ill for more than a month, and was much reduced by repeated attacks of fever, which at first assumed the character of the infantile remittent, apparently depending on disordered state of the bowels, which were very torpid. After this state of the alimentary canal had been corrected the fever continued to recur—at first in a tertian, but latterly in a quotidian form, with morning paroxysms which lasted till after noon, having the cold, hot, and sweating stages distinctly marked. She took, in the whole, fourteen grains of the sulphate of quinine, in doses of one grain twice a day. She had no return of the paroxyms.

The second case was a patient of Mr. Henderson's, a girl upwards of seven years of age, who had been attacked ten days before with a quotidian intermittent strongly marked. She was cured by six grains of the sulphate of quinine. Some other cases are related, in which the medicine evinced similar effects.

Dr. Dickson has prescribed the remedy with much advantage, as a tonic and stomachic, in some other cases ; and dwells, with

* After the extraction of the quinina, the yellow bark is as tasteless as so much wet saw-dust.

justice, on the great benefit which is likely to result from having a medicine of such power concentrated in such a small space.

In respect to our own experience, we can testify to the powers of this medicine in many diseases of a periodical or recurrent nature, and we had lately an instance where its virtues were exhibited in a very decided manner, and where, from gastric irritability, there was every reason to believe that no other preparation of bark could have been taken in sufficient quantity to arrest the progress of the disease.

A gentleman of rank left town for his country seat in Cheshire, in the beginning of May, of the present year. He attended the races at Chester a few days after his arrival in the country, and was there exposed to the cold easterly winds, at that time prevalent. A few days after the races were over, he was seized with deadly cold chills, rigors, head-ache, pains in all his limbs, and sickness at stomach. Being of a very delicate and weakly constitution, these symptoms were very alarming, as the pulse fluttered, and sometimes entirely left the wrist, while his whole appearance was most ghastly. These phenomena, after a time, ended, not in reaction, but in cold clammy perspirations, if possible more alarming and distressing than the preceding cold and dry stage. Several hours were passed in this way, when a natural heat very slowly and gradually returned, and the patient continued languid, but free from complaint during the succeeding day. On the morning of the third day he awoke with a pain over the left eye-brow, and two hours later than the period of the former attack, the same paroxysm of cold shivering and cold sweating stages ensued. Four of these attacks had taken place, each preceded by the ominous pain over the eyebrow—each happening on alternate days, and each occurring at a later period, by two hours, than the preceding, when the writer of this article was summoned from town, and arrived on the evening of the apyrexial day. The patient was lying on his sofa, languid and debilitated, with very little appetite, feeble pulse, and blanched altered countenance. On learning the preceding history, it was concluded that the disease was an imperfect ague, and that the want of the hot stage was merely dependent on want of energy in the constitution. The sulphate of quinine was instantly procured from Chester, and ordered in two grain doses every four hours, while cordial draughts of confectio aromatica, carbonate of ammonia, tincture of cinchona, &c. were prescribed at similar intervals. Next morning was ushered in with the precursory pain over the left eye, and exactly as the clock struck the hour, the paroxysm came on. It differed only in degree from those already described—and it was shorter in duration. But even in this mitigated form, it was of a very formidable character. For more than three quarters of an hour little or no pulse could be felt in any tangible artery—the skin was of a corpse-like coldness—the mind was wandering—the respiration hurried, short, and the breath expired was actually cold to the hand. There was a universal tremor over the body, with pains and cramps through the limbs and along the spine, with gastric irritability. It ended, as usual, in cold, clammy perspirations, after which the patient fell into a troubled interrupted sleep, and next morning complained only of excessive debility. He was obliged to be wheeled on a sofa from his bed room into one of the sitting rooms this day. The sulphate of quinine and the cordial draughts were now pushed on without interruption, day and night, and he never had another paroxysm. He gradually, but pretty rapidly recovered strength, and in a few days was able to ride round his park.

It is proper, before closing this paper, to observe that two or three practitioners on the Continent have published accounts of the injurious effects of sulphate of quinine. We are not much disposed to wonder at this, considering how timid our continental brethren are in administering purgatives and other evacuants in either acute or chronic complaints;—consequently, the exhibition of this highly powerful tonic, without due preparation, where there are visceral obstructions or disordered secretions, must be expected to be attended with bad effects. Thus Dr. A. Menard has lately published a paper in the November Number of the Revue Medicale, wherein he states that several cases have occurred to him, or come under his observation, where the paroxysms of the disease (intermittent and remittent fevers) had been speedily cut short by the exhibition of this salt, in large doses—but, after several days, the disease had sometimes returned, and attacks of inflammation or engorgement of the liver or spleen, had frequently supervened to the sudden arrest of the febrile paroxysms. He has observed these visceral inflammations and obstructions to have been so often followed by dropsical affections, that he considers these last diseases to have become more common since this remedy has been generally employed. Some other French writers* have also noticed affections of the mucous membrane of the bowels following large doses of the sulphate of quinine. We do not apprehend such consequences in this country, where more attention is paid to sanguineous and intestinal evacuations.

* Dr. E. Desportes, for example, in Revue Medicale for December last.

We must not pass over entirely unnoticed, the memoir of Dr. Martinet. This gentleman practised in Italy, in the years 1821–2, at Migliarino, a marshy plain near Pisa, and where intermittent and remittent fevers were but too plentiful. His patients were principally peasants, in the prime of life. He first tried comparatively small doses, as ten or twelve grains during the apyrexia; but in this quantity he made no impression on the paroxysms. He then administered from 18 to 24, or 30 grains in the same period, and checked the fever, generally after the first paroxysm. In some cases, of long standing, he was obliged to carry the quantity to 35 grains in the interval of fever. In no instance did he observe any bad effects from the remedy. In some instances, there were symptoms originally of gastro-intestinal irritation or inflammation, and in these he abstained from the remedy till the proper steps were taken for removing the said affections. At Florence, our author pursued the same practice, and with similar success, as also at Rome. He has detailed a number of cases, but these we need not cite.

